# Effect of design and surgical parameters variations in mobile‐bearing versus fixed‐bearing unicompartmental knee arthroplasty: A finite element analysis

**DOI:** 10.1002/jeo2.70053

**Published:** 2024-10-26

**Authors:** Thomas Luyckx, Edoardo Bori, Rachele Saldari, Sara Fiore, Virginia Altamore, Bernardo Innocenti

**Affiliations:** ^1^ Department of Orthopedic Surgery AZ Delta Roeselare Belgium; ^2^ BEAMS Department (Bio Electro and Mechanical Systems), École Polytechnique de Bruxelles Université Libre de Bruxelles Bruxelles Belgium

**Keywords:** design parameters, fixed bearing, mobile bearing, sensitivity study, UKA

## Abstract

**Purpose:**

Unicompartmental knee arthroplasty (UKAs) are available in the market as fixed‐ and mobile‐bearing (FB and MB) and can be characterised by a different set of design parameters in terms of geometries, materials and surgical approaches, with overall good clinical outcomes. However, clear biomechanical evidence concerning the consequences of variations of these features on knee biomechanics is still lacking; therefore, the present study aims to perform a sensitivity analysis to see which outcomes are affected by these variations.

**Methods:**

For both MB‐UKA and FB‐UKA, five design and surgical parameters were defined (bearing insert thickness, tibial component material, implant components friction coefficient, antero‐posterior slope angle and level of tibial bone resection). Two control models were defined based on standard configurations for both implants. Finite element analysis was chosen to perform this study, and different parameter combinations (216 models in total) were implemented and tested at both 0° and 90° of flexion, using a previously validated finite element knee model. The results were then evaluated in terms of bone and polyethylene Von Mises stress and tibio‐femoral contact area.

**Results:**

Bearing thickness, tibial bone cut and slope angle were found to be the most sensitive parameters for both types of UKAs. Specifically, changes in these parameters in the FB‐UKA appeared to induce more significant variations in the polyethylene insert (both in terms of polyethylene stress and contact area), while in the MB‐UKA, these changes influenced bone stress distribution more.

**Conclusions:**

Surgical parameters returned to have a more significant influence than material and friction variations; furthermore, the outcomes most affected by parameter variations were the insert‐related ones for FB‐UKA while for the MB‐UKA were the ones regarding tibial bone stresses.

**Level of Evidence:**

Not Applicable.

**Abbreviations**
ACL: anterior cruciate ligament
aMCL: anterior medial collateral ligament
AP: antero‐posterior
CoCrMo: cobalt‐chromium‐molybdenum
CT: computed tomography
E: elastic modulus
FB: fixed bearing
ISO: International Organisation for Standardisation
LCL: lateral collateral ligament
MB: mobile bearing
PCL: posterior cruciate ligament
pMCL: posterior medial collateral ligament
PMMA: poly‐methyl methacrylate
ROI: region of interest
Ti6Al4V: titanium‐aluminium‐vanadium
TKA: total knee arthroplasty
UHMWPE: ultra high molecular weight polyethylene
UKA: unicompartmental knee arthroplasty

## INTRODUCTION

Unicompartmental knee arthroplasty (UKA) has emerged as a valuable alternative to Total Knee Arthroplasty for the treatment of antero‐medial osteoarthritis (OA) [2, 70]. This procedure involves replacing the damaged compartment while preserving bone stock and cruciate ligaments, resulting in minimal invasiveness, reduced blood loss, faster recovery time, improved functional recovery, restoration of native knee kinematics and range of motion and simplified future revision scenarios [7, 53, 58, 65]. Overall, registries report a survivorship rate of 85%–90% at 10 years after surgery UKA [6, 53, 58, 64]. The associated revision rate is, therefore, higher than the one found for TKA [49], and the main reasons leading to these failures are OA progression in the contralateral side [6, 9, 48], loosening of the components [6, 41, 47] and malpositioning of the prosthetic components, which leads to high stress on soft tissues, knee pain and eventual failure of the implant‐bone fixation due to bone overload [7, 10, 17, 61].

Currently, the available UKA models can be grouped into fixed‐bearing (FB) and mobile‐bearing (MB) designs [33]. The crucial distinction between these designs lies in how the tibial component and insert interact: fixed‐bearing UKA is designed as a resurfacing procedure, with the insert rigidly constrained to the tibial tray and having a non‐congruent surface with respect to the femoral component; mobile‐bearing models involve instead the capability of the insert to slide on the polished tibial component, and the interface between insert and femoral component presents higher congruency. This mobile insert is commonly referred to as the “meniscal bearing”.

It is thus important to address the fact that these different designs involve consequently different features and parameters in terms of material and component dimensions, and these variations may have a significant impact on the biomechanical outcomes of the implant. As an example, to accommodate a mobile bearing, the tibial component requires a specific low friction coefficient and therefore is usually made of a cobalt‐chromium alloy; [52] however, this material presents higher elastic modulus compared to the titanium used in fixed bearing designs and this choice could affect how joint stresses are transmitted to the bone. A stiffer material may indeed result in stress shielding, with the load not adequately transferred to the bone [20, 37, 59], and potentially lead to the generation of the radiolucent lines clinically found often associated with the loosening of the component [37].

On the other hand, a fixed bearing design will involve higher relative displacements between the femoral component and insert, due to their non‐congruent interface: this can then lead to a higher wear rate of the insert over time, thus potentially affecting the performance and longevity of the implant [20, 52, 59]. The mobile bearing insert design, characterised by higher insert congruency, does not imply this amount of relative displacement with the femoral component but, at the same time, involves an additional sliding interface between the insert and the tibial component, which could in turn increase the wear process [32, 33]. However, it is worth mentioning that the introduction of crosslinked polyethylene in the latest years had a remarkable role in mitigating these wear‐related issues, which currently represent only a marginal cause of failure [46, 68].

It is therefore crucial to consider a broad series of factors when selecting the appropriate design for a patient, taking into account the trade‐offs between mobility, stress distribution, wear and potential complications associated with each parameter to ensure optimal biomechanical performances and long‐term success of the implant. To assist surgeons in the decision‐making process, biomechanical guidelines are of paramount importance; therefore, this study aims to investigate the impact of surgical and design parameter variations for both fixed‐ and mobile‐bearing UKA. To perform this multi‐factor comparative analysis, the finite element model is the best approach to achieve the highest comparative value.

## MATERIALS AND METHODS

This study is structured as follows:

1) Definition of the addressed clinical and design parameters, and definition of the range of variability for each parameter;
2) Definition of the parameters corresponding to the two ideal configurations for FB and MB designs;
3) Implementation of three‐dimensional models defined in point 2 (used as control models);
4) Starting from the control models, development of the different UKA configurations according to the parameters defined in point 1;
5) Biomechanical in‐silico simulations of the different configurations implemented in points 3 and 4;
6) Analysis of the results, in terms of comparison of the UKA performance, sensitivity study and statistical analysis both at the tibio‐femoral and at the implant‐tibial bone interface.

The presented model was developed following the approach of previously validated knee finite element models, focused on the implant of TKA and UKAs [3, 14, 35, 36, 39].

The approach applied involved the implementation of the following features.

### Geometries and material

A simplified version of the joint model was used for the present study; it consisted of the proximal section of the tibia [66], along with the ligamentous structures in charge of the knee stability, including the anterior and posterior cruciate ligaments and the medial and lateral collateral ligaments. For the bone geometry, the physiological 3D tibial bone model was reconstructed from computed tomography (CT) images of a 43‐year‐old woman [39]. This model includes cortical bone, cancellous bone, intramedullary canal and articular cartilage.

Two prosthesis designs were considered for the study to represent the fixed‐bearing and mobile‐bearing UKAs. The models implemented are based respectively on the ZUK Uni, (Lima Corporate) and the Oxford Partial Knee (Zimmer‐Biomet Inc.). The 3D models were then developed by virtually implanting the prostheses in the bone; to guarantee the possibility of comparing the outcomes of the two UKAs, accurate measurements were realised in the patient bone, and corresponding sizes for the two designs were selected. The proper size was furthermore checked by a senior surgeon (TL) with more than 15 years of experience in UKA surgery.

For the mechanical properties modelling, materials involved in this study were considered linear elastic [39]. The tibial bone was modelled implementing a distinction between cortical and cancellous bone [42], with the cortical bone modelled as transversely isotropic (with the principal axis corresponding to the mechanical axis of the tibia itself) and the cancellous bone as isotropic. The properties of the prosthesis material were assumed as isotropic and taken from the literature [3]. The values used can be found in (Table 1).

**Table 1:** Material properties used for this study.

Part	Material	Elastic modulus	Poisson's ratio
Femoral and mobile bearing tibial components	CoCrMo	E = 220 GPa	0.3
Fix bearing tibial component	Ti6Al4V	E = 110 GPa	0.3
Insert	UHMWPE	E = 0.685 GPa	0.4
Tibial Bone	Cortical bone	$E_{1}=$ 11.5 GPa $E_{2}=$ 11.5 GPa $E_{3}=$ 17 GPa	$\nu_{12}=$ 0.58 $\upsilon_{23}=$ 0.31 $\upsilon_{31}=$ 0.31
	Cancellous bone	E = 2.13 GPa	0.3

*Note*: For the cortical bone, the direction 3 represents the axial direction. The plane 12 represent the plane orthogonal to the direction 3.

Frictional contact interaction was implemented between the prosthetic components and the insert, with a friction coefficient varying from the control value of µ = 0.05 according to the parameter configuration (as detailed below). For the implant‐bone interaction, a tangential behaviour was adopted, and the coefficient of friction was assumed as the one between bone and PMMA (µ = 1 [62]), to simulate on the bone the effects of a cemented implant.

Lateral and medial (divided into an anterior and posterior bundle) collateral ligaments and anterior and posterior cruciate ligaments were modelled as beams with Elastic Modulus, cross‐sectional area, and pre‐strain values defined in agreement with previously validated studies in the literature [14, 28, 35, 36, 39], as reported in Table 2.

**Table 2:** Ligament properties used for this study.

Ligament	Young's modulus [MPa]	Poisson's ratio	Initial Strain ε_r_	Cross‐sectional area (mm^2^)
LCL	345	0.45	0.08	18
aMCL	332	0.45	0.04	25
pMCL	332	0.45	0.04	25
ACL	169	0.45	0.08	42
PCL	177	0.45	0	60

As the focus of the analysis is the effects on the UKA components and the tibial bone, the femur and the fibular bone were not taken into account in the present work. Furthermore, since the prosthetic system under analysis mainly addresses the tibio‐femoral joint, the patella and the relative ligamentous structures were not taken into account in the model.

### Parameters and configurations

Five design parameters were identified and taken into account for the present study: the thickness of the bearing insert, the material (and relative mechanical properties) of the tibial component, the friction coefficient between the implant components, the antero‐posterior slope angle and the level of the tibial bone resection. For each prosthesis, a control model was thus defined based on the standard design features for the different implants (see Table 3).

**Table 3:** Control configurations for FB and MB UKA designs.

	Insert thickness (mm)	Tibial component material	Friction coefficient	A‐P slope angle (°)	Tibial bone cut (mm)
Fixed bearing	6	Ti6Al4V	0.05	5	0
Mobile bearing	7	CoCrMo	0.05	7	0

Each parameter was then modified among a range of variations to define and implement all the altered models. Three different thickness values were chosen for the insert: the most commonly implanted (6 mm for the FB and 7 mm for the MB [29]), a thicker insert (+1 mm) and a thinner one (−1 mm).

The tibial component materials considered for the study were a CoCrMo alloy (commonly used for the MB design) and a Ti6Al4V alloy (typically used for the FB design).

The dynamic friction coefficient used was 0.064–0.15 for CoCrMo−UHMWPE contact and 0.07–0.16 for Ti‐64–UHMWPE contact [63]. The approximated limits of these ranges (0.05–0.2) were chosen to simulate two different conditions of lubrication [8], which usually depend both on the patient and the materials.

Different antero‐posterior slope angles are suggested by manufacturing companies. For the MB design, a slope angle of 7° ± 5° is suggested [72], while for the FB it is stated at 5° ± 2° [73]. To evaluate the influence of this feature on the biomechanics of the joint, three different slope angles were considered: 3°, 5° and 7°.

The level of the performed tibial bone transversal cut is a crucial parameter since it can determine a condition of understuffing or overstuffing of the joint [31]. Based on that, the design configuration with the theoretical correct cut was considered [72, 73], along with a 2 mm deeper cut and a 2 mm shallower cut.

### Assembly and boundary conditions

The different UKA configurations defined were then implanted in the respective knee joint models, following the surgical indications as reported by the manufacturer [72, 73].

Correct mutual positioning of the implant elements was achieved following the data found in the literature [21, 30, 55, 74].

The distal extremity of the tibia was constrained in all directions; the load applied, equal to three times the average body weight, was proportionally split between the medial and lateral compartments according to what was specified in the literature [24, 44, 69]. The application points were located following the ISO standard 14243‐1 [40], indicating the procedure to identify the flexion/extension axis. The joint was tested both in full extension (0° of flexion) and at 90° of flexion.

### Regions of interest and outputs

The outputs addressed in this comparative study were defined as the stress magnitude in both the UKA polyethylene insert and the tibial bone, as these values cover a fundamental role in determining implant performances.

**Figure 1:**
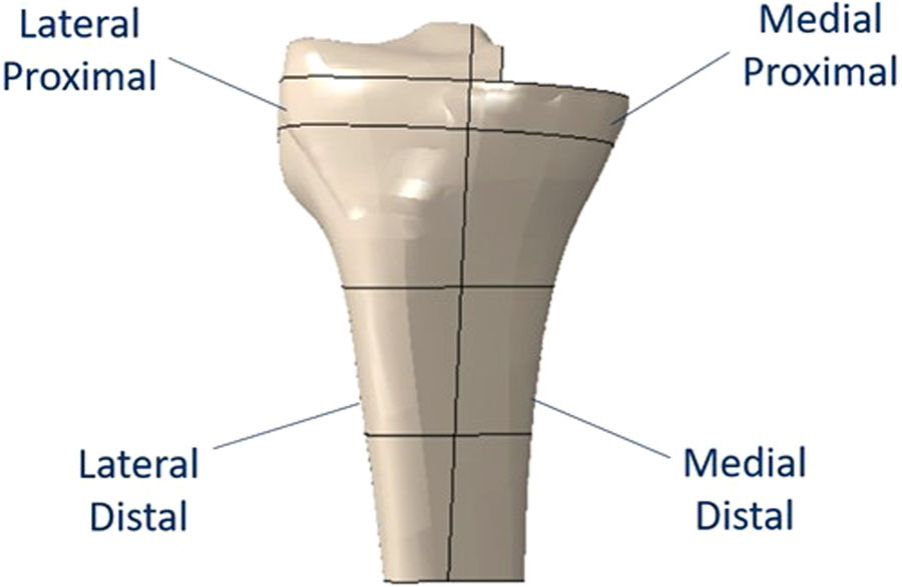
Regions of interest in the bone.

To analyse in detail the stress distribution in the bone, four regions of interest (ROIs) were defined on the tibia: two local regions, close to the tibial proximal surface (Medial Proximal ROI and Lateral Proximal ROI, investigated with a depth of 10 mm starting from the transversal tibial cut level), and two distal regions (medial distal ROI and lateral distal ROI, 30 mm thick starting from the proximal regions) (See Figure 1). This choice is also in agreement with previous in‐silico studies on TKA and UKA [35, 37, 38, 39].

The values taken into consideration and extracted after each test were the following:

– The average Von Mises stress on the polyethylene component;
– The average contact area between the femoral component and the UHMWPE insert;
– The average Von Mises stress on the proximal medial ROI;
– The average Von Mises stress on the proximal lateral ROI;
– The average Von Mises stress on the distal medial ROI;
– The average Von Mises stress on the distal lateral ROI;

For both flexion angles, the results of the fixed‐ and mobile‐bearing control models were then taken as a reference and compared to the outcomes of all the other configurations. Since the primary objective of this study focuses on the analysis of the variations of the output according to the different parameters configuration, the deviations of these outputs from the ones obtained from their relative control models were considered to be the most representative values to address; these results were then expressed as percentages to simplify their interpretation. This approach was chosen as it allowed us to evaluate easily which outputs were most sensitive to variations of the parameters, and which parameters were the ones influencing them in the most significant way. Consequently, it was possible to identify which UKA bearing was more sensitive to specific parameter changes and determine which output was most influenced as a result.

### Finite element analysis

Tetrahedral quadratic elements with a variable dimension were used to define the mesh for both the UKA elements and the tibial bone. The meshing of the four tibial ROIs was performed with a mesh size of 1, while the remaining part of the bone was meshed more coarsely (size = 5) to reduce the simulation times.

Starting from the control configurations, the first test was carried out and convergence of the model was performed. Once the setup was defined, all the remaining simulations were then executed.

Abaqus/Standard version 2019 (Dassault Systèmes) was used to perform all the finite element simulations.

## RESULTS

**Figure 2:**
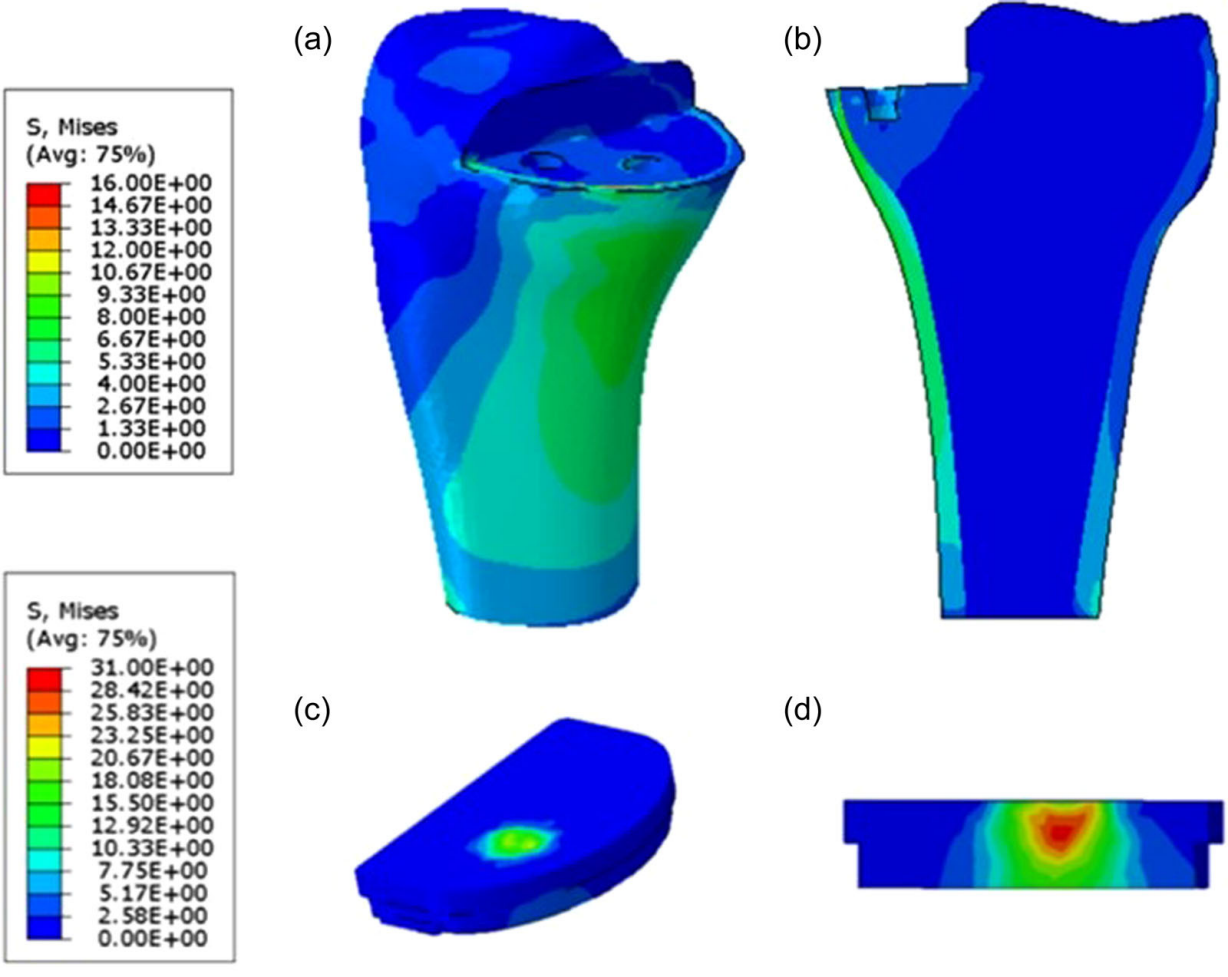
Qualitative overview of the average Von Mises stress distribution on the tibial bone (a), on the frontal section of the tibial bone (b), on the UHMWPE insert (c) and the frontal section of the UHMWPE insert (d).

Figure 2 depicts a qualitative overview of the Von Mises stress induced on the full‐extension FB control model. It can be observed how the bearing component presents a stress peak located at a non‐superficial point, close to the contact area (in accordance with Hertz's Theory regarding contact mechanics). Comparing the Von Mises stress distribution between the full extension and the 90° of flexion configurations for both FB and MB control models, analogous patterns were found.

Among the analysed parameters, bearing thickness, tibial bone cut and slope angle returned to be parameters to which both FB and MB implants are the most sensitive.

**Figure 3:**
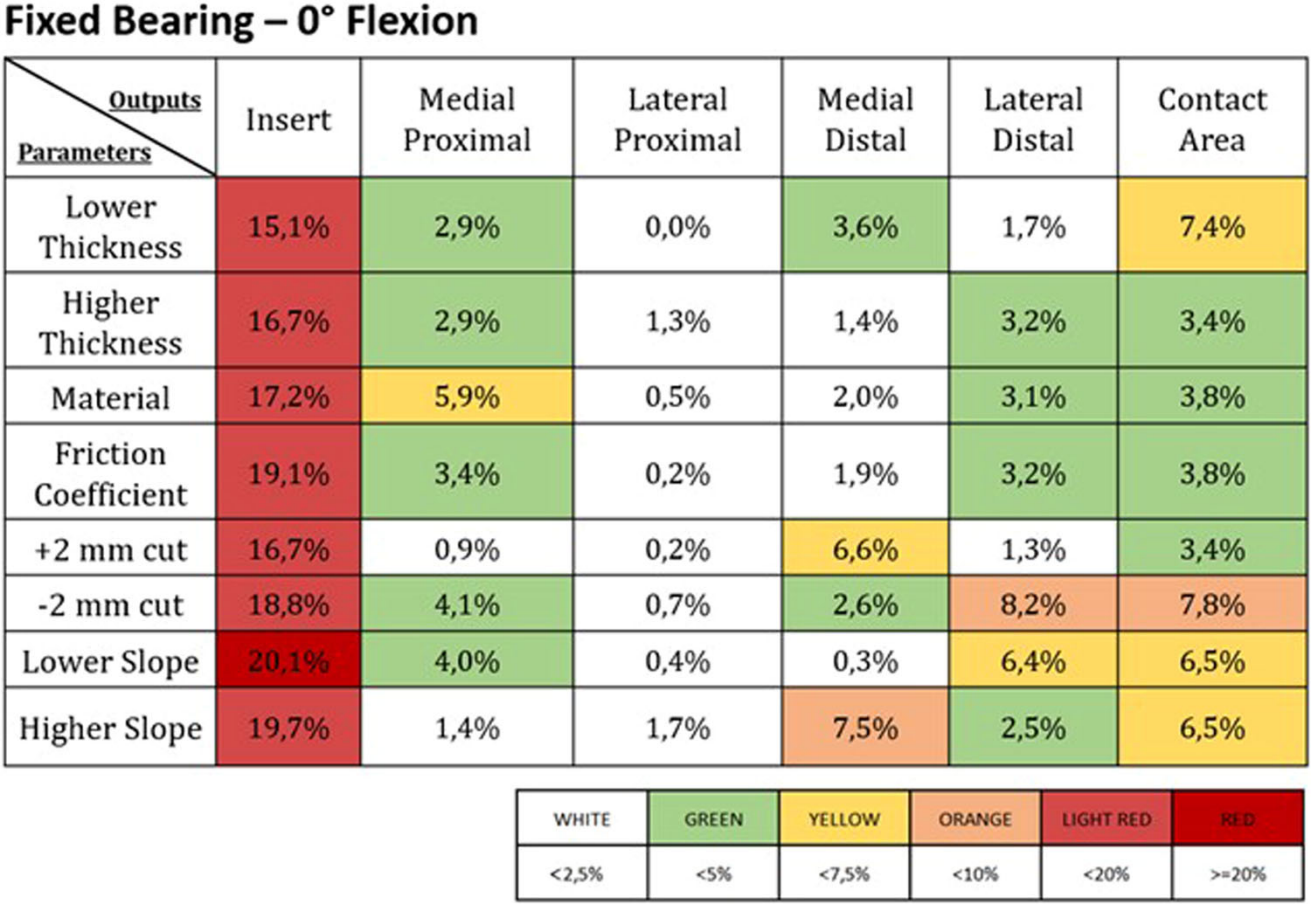
Percentage change from the control values in the different outputs (listed in the first line) induced by the different parameters (reported in the first column) in a fixed bearing UKA at 0° of flexion. The values of the percentage changes are highlighted with different colours based on the legend found below.

**Figure 4:**
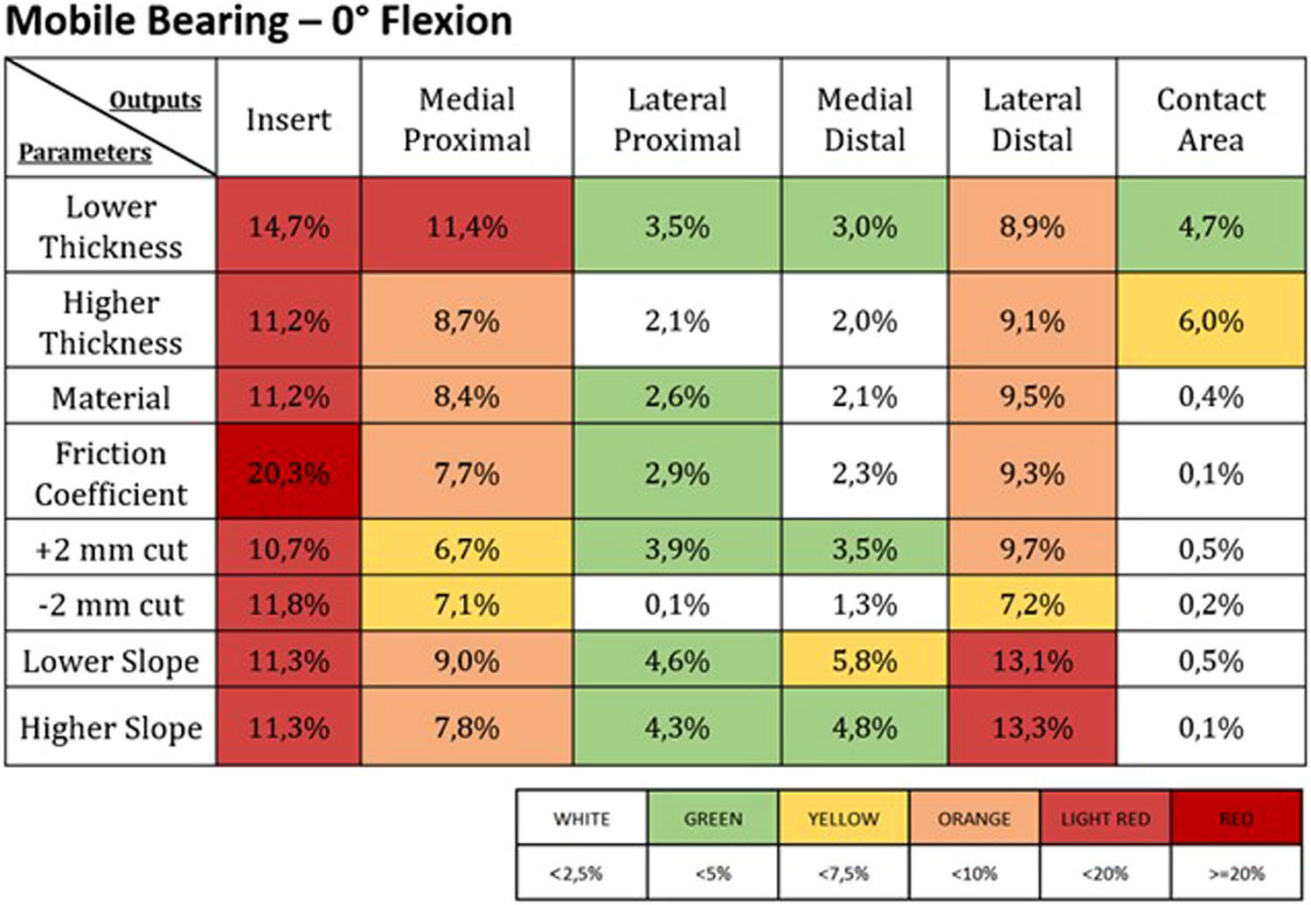
Percentage change from the control values in the different outputs (listed in the first line) induced by the different parameters (reported in the first column) in a mobile bearing UKA at 0° of flexion. The values of the percentage changes are highlighted with different colours based on the legend found below.

**Figure 5:**
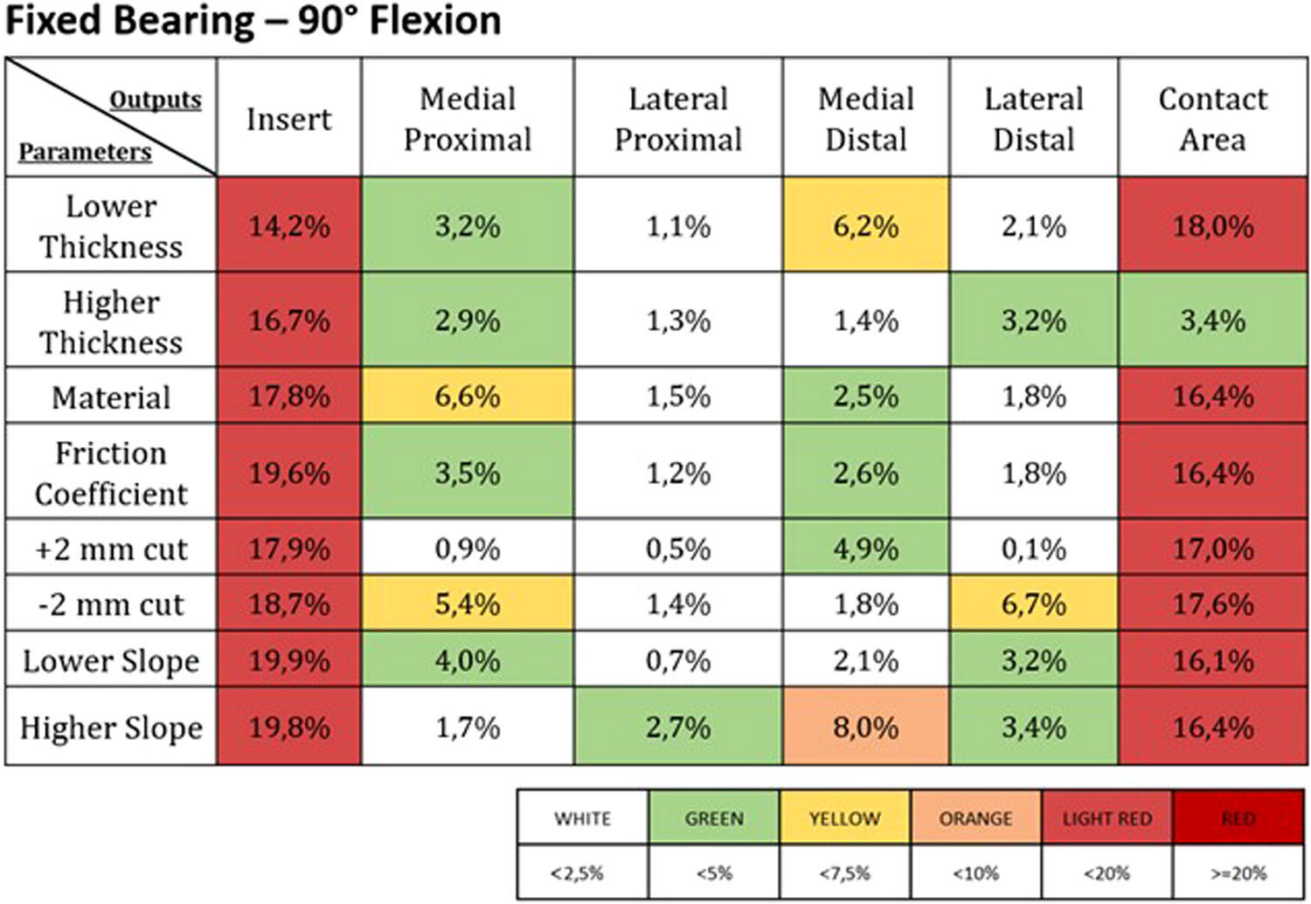
Percentage change from the control values in the different outputs (listed in the first line) induced by the different parameters (reported in the first column) in a fixed bearing UKA at 90° of flexion. The values of the percentage changes are highlighted with different colours based on the legend found below.

**Figure 6:**
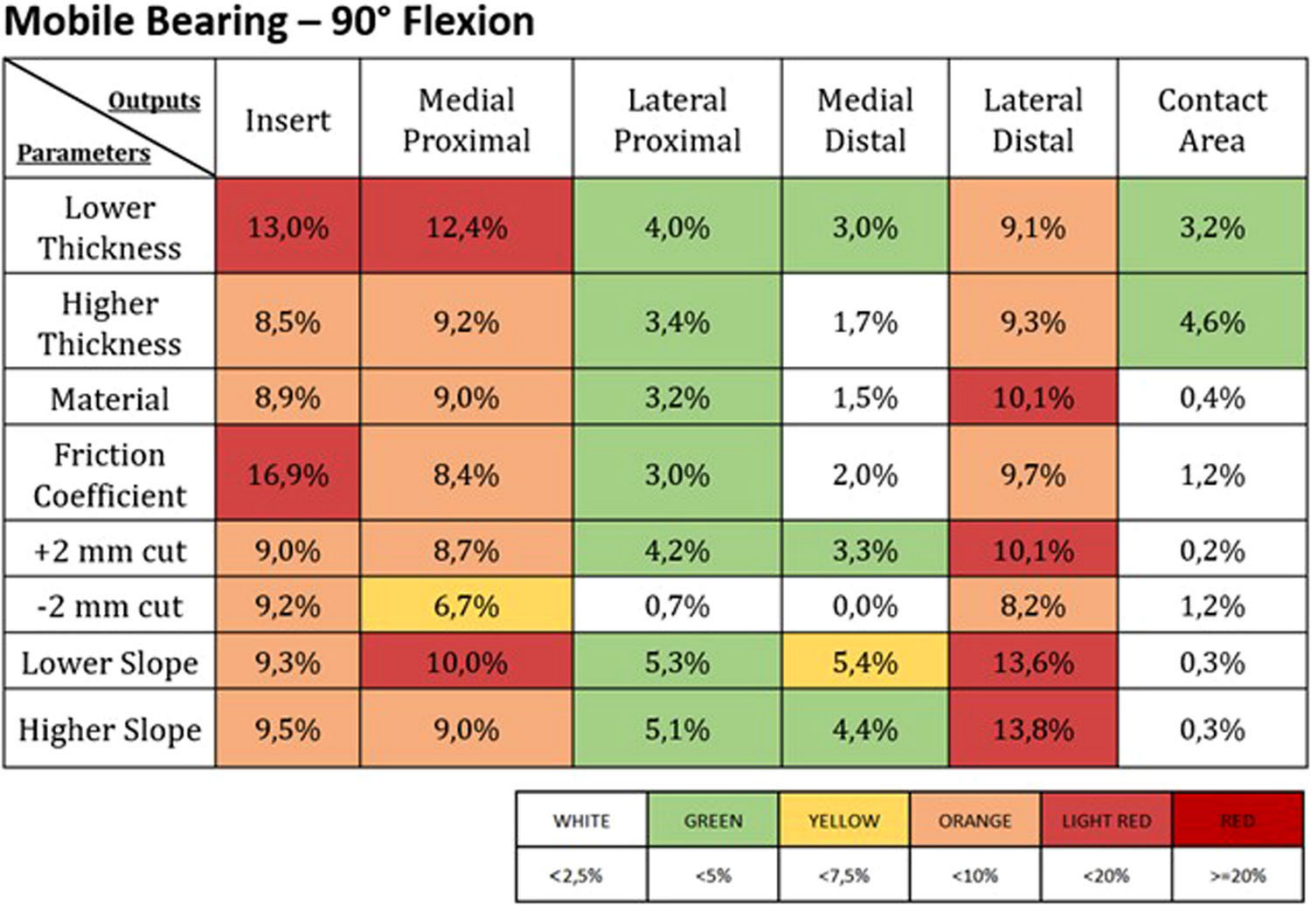
Percentage change from the control values in the different outputs (listed in the first line) induced by the different parameters (reported in the first column) in a mobile bearing UKA at 90° of flexion. The values of the percentage changes are highlighted with different colours based on the legend found below.

Figures 3, 4, 5, 6 report the deviations from the control induced by the different parameter changes in the FB and the MB UKAs; these results were expressed as percentual variations from the control values for each of the different outputs addressed, and a colour map was used to highlight the different levels of deviation.

Overall, the variations in insert‐related outputs were found to be more sensitive in the fixed‐bearing design, while in the mobile‐bearing design, they remained more consistent. This difference is likely due to the distinct polyethylene insert designs—flat for the FB and congruent for the MB UKA. However, regarding tibial bone outputs, the mobile‐bearing design was more sensitive to parameter variations this time.

**Figure 7:**
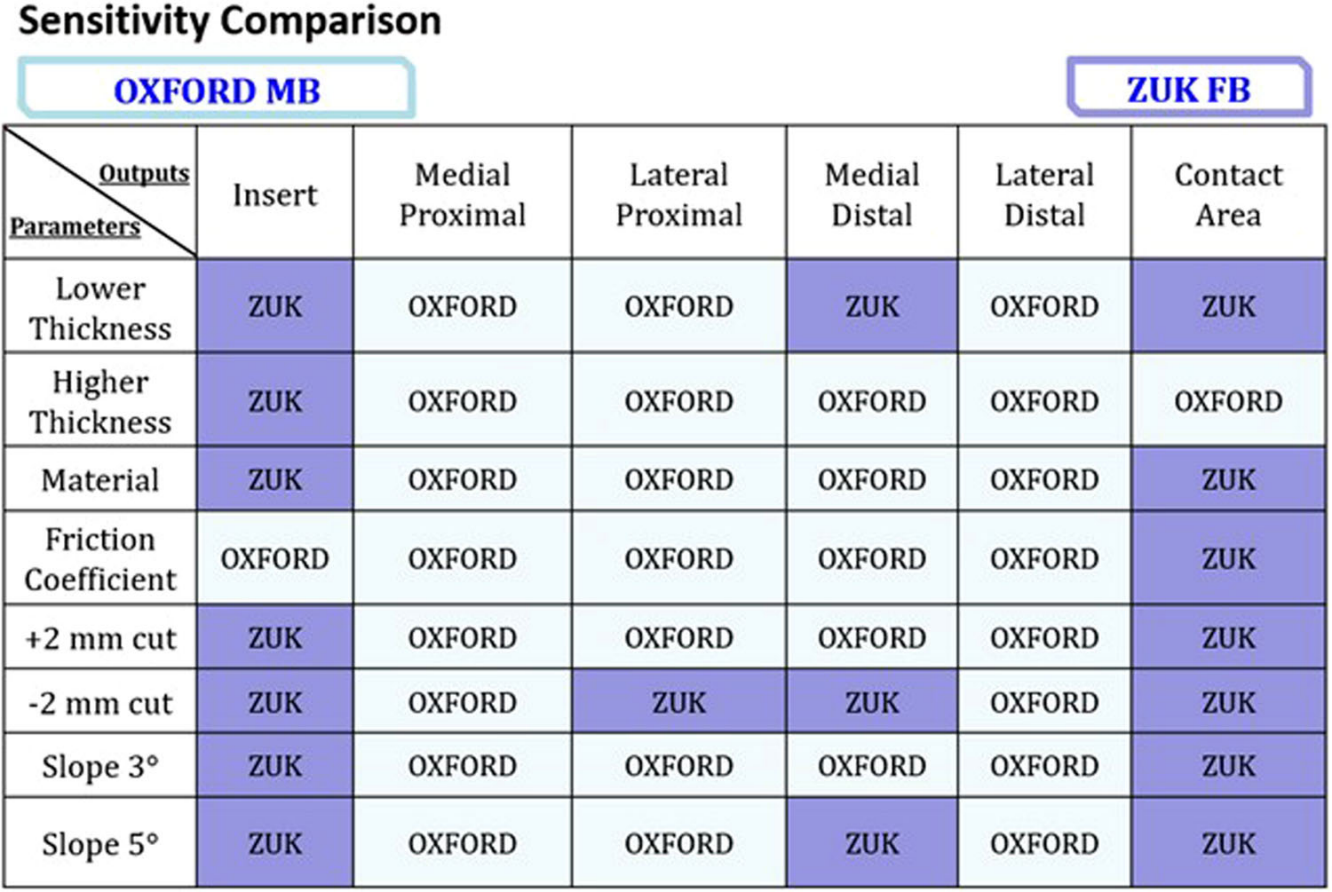
Sensitivity comparison of an OXFORD MB and a ZUK FB UKA considering the different outputs (listed in the first line) induced by the different parameters (reported in the first column).

These above‐mentioned considerations can be further appreciated in Figure 7, which reports the most sensitive design (between the OXFORD MB and ZUK FB implants) for each combination of outcome and parameter alteration. Indeed, from this picture, it can be noted that changes in the surgical and design parameters in the MB implant induced more significant output variations when addressing the ROIs on the tibial bone, while the FB implant returned to be more sensitive in terms of insert‐related outcomes.

## DISCUSSION

The key finding of this study indicates that the FB design, when surgical or implant design variations occur, is able to return outcomes in terms of tibial stress distribution that are closer to the ones obtained in the ideal control configuration when compared with the relative ones obtained with the MB design. On the other hand, the outcomes relative to the polyethylene insert of the MB design demonstrated lower sensitivity to design and surgical variations compared to the one of the FB design. Overall, the FB design appeared to provide more robust results for bone stress distribution, while the MB design returned to do the same for the implant components instead.

When assessing the overall survival rates of mobile‐bearing and fixed‐bearing designs through registries, no discernible disparity is evident between the two: for instance, several [19, 51, 56] meta‐analyses failed to identify an eventual superiority between FB or MB UKA implants in patients with monocompartmental knee OA, while, on the contrary, Burger et al. [12] concluded that MB reported a greater revision rate compared to FB implants but similar clinical outcomes, in a systematic review including 2265 procedures (28 studies). Moreover, a common observation concerning the knee implanted with an MB design is the presence of radiolucent lines [37], which may suggest a potential abnormal loading of the bone. Intriguingly, such radiolucent lines are not observed with the FB design. Our research findings, indicating that the MB UKA outcomes are indeed more susceptible in terms of bone stress variations in case of deviation from the ideal parameters, offer insight into this phenomenon, potentially elucidating the underlying reasons for this distinction.

Irrespective of design, tibial bone stresses notably vary depending on whether the tibial component is fabricated from CoCrMo or titanium. The use of the stiffer alloy, indeed, reduces the stresses on the medial proximal tibia, in contrast to scenarios involving a titanium tibial component: the latter design induced bone stresses closer to physiological conditions, adding a further possible explanation for the aforementioned radiolucent lines observation in case of MB UKAs, which involve the use of CoCrMo.

A further difference between the two designs can then be found in terms of surgical technique; in the case of MB design, the reference for the depth of tibial resection is taken from the posterior femur [18, 23, 43, 54, 67]. This factor can lead to slightly higher values of tibial bone resection because the cartilage on the posterior femur remains intact (with an average thickness of 2 mm) in cases of antero‐medial OA, and this theoretically leads to an additional 2 mm of tibial bone resection. On the other hand, the surgical technique for tibial resection in FB design is based on the extension gap principle [67]; it is furthermore worthy to mention that in cases of bone‐on‐bone gonarthrosis, a tendency to remove less tibial bone is reported, and the combination of these factors can therefore lead to differences in the level of resection, which finally play a crucial role in determining the stress distribution within the bone.

Concerning the surgical factors examined, the analysis identified the tibial slope as the most significant determinant of stress level variation in the tibial bone. Consequently, caution is advised when dealing with excessive tibial slope. This recommendation is supported by surgical observations, indicating that a combination of heightened slope, deeper tibial resection and compromised bone strength may contribute to early loosening of the tibial component [4]. Moreover, the results align with several studies on the improvement of patient outcomes when an accurate implant position is achieved. For instance, Zambianchi et al. found higher survival rates and good‐to‐excellent clinical outcomes at a minimum 10‐year follow‐up in 239 patients with UKA implanted using robotic surgery [71]. Similarly, the prospective multicenter study by Bayoumi et al. [5] reported improved 10‐year survivorship and patient satisfaction following robotic‐arm‐assisted medial unicompartmental knee arthroplasty. Additionally, Fossey et al. [27] identified that robotic assistance in medial UKA patients was associated with better accuracy in tibial implant positioning, post‐operative limb alignment and joint line restoration, which translated to improved survival at mid‐term follow‐up in the robotic group. The findings also concur with those of Micicoi et al. [50], who demonstrated in a retrospective study of 144 medial UKA patients from 2015 to 2020 that functional outcomes after medial UKA can be influenced by implant alignment in the coronal plane.

An additional observation revealed significant differences not only in stress levels within the medial proximal tibial bone but also in the medial distal bone and even the lateral tibial bone. This discovery represents a novel contribution to the field, as previous studies [22, 38, 39, 60] have predominantly concentrated on the medial proximal tibial region neglecting the outcomes in the distal regions.

However, this study has some limitations, mainly related to the numerical nature of this approach. Indeed, the material models used are all assumed to be linearly elastic; this is a significant assumption, which however allows to achievement of a proper approximation of the mechanical properties and facilitates the comparison between different configurations; it is therefore typically used for this kind of analyses in the literature [1, 11, 14, 15, 34, 38, 39, 57]. The finite elements model implemented is thus not able to fully capture the complexities of in‐vivo biomechanics, and only considered the bone geometries of a single patient; moreover, the fact of having considered only static boundary conditions does not allow an analysis of long‐term effects or fatigue. However, it should be noted that this study does not directly address these topics but rather focuses on a comparative analysis of different configurations. By using the same overall model, characterised by identical bone geometries, assumptions and approximations, the study directly correlates outcomes with the parameters modified from the control model. This approach was therefore chosen as it aligns with the study's objective, although analysing multiple patients (with different geometries and different degrees of severity) could provide additional insights.

The overall validity of the results is therefore based on the previously validated numerical modelling approach [3, 14, 16, 35, 36, 39] and on the consistent application of the same assumptions across all compared models, ensuring that the results are robust and reliable within the scope of the study, given the assumptions made.

Before this research, few literature studies adopted finite element analysis to investigate and compare the effects of insert contact pressure and stress for FB and MB UKA. In detail, Kwon et al. [45] reported the same tendency of the MB design to induce lower insert stresses and contact pressures than the FB design, suggesting that the MB UKA, by lowering the stress on the opposite compartment, can reduce the overall risk of progressive knee OA. These results can be assumed as a consequence of the higher degree of congruency between the articular surfaces in the MB design, which mimics how the meniscal bearings reduce surface contact stress [13, 26]. The abovementioned evaluations are in line with Emerson et al. [25]; they observed that the higher failure rate in FB UKA was due to polyethylene insert wear.

## CONCLUSION

The results obtained in this study demonstrate that, for each UKA design, small variations of the parameters from the ideal configuration induce variations in both insert and bone stresses when compared to the control configuration. Notably, Fixed Bearing designs resulted in lower variations in bone stress, whereas Mobile Bearing designs ensured more consistent values for the insert. Surgeons should consider these findings when determining the suitable design for individual patients, selecting the most appropriate option based on the patient's situation and critical regions. For instance, if the quality of the proximal tibial bone suggests potential risks of failure, the surgeon should consider opting for a design with minimal variability in this region, such as the Fixed Bearing design. Conversely, if concerns primarily revolve around the tibio‐femoral interface and insert wear, the Mobile Bearing design may represent the preferable choice.

Such considerations can then be crucial for optimising patient outcomes and minimising potential complications and failures that can be associated with implant design.

## AUTHOR CONTRIBUTIONS

*Conceptualisation*: Thomas Luyckx and Bernardo Innocenti. *Methodology*: Edoardo Bori, Rachele Saldari and Bernardo Innocenti. *Formal Analysis*: Edoardo Bori, Virginia Altamore and Sara Fiore. *Investigation*: Thomas Luyckx, Rachele Saldari, Edoardo Bori, Virginia Altamore, Sara Fiore and Bernardo Innocenti. *Data Interpretation*: Thomas Luyckx, Edoardo Bori and Bernardo Innocenti. *Writing—original draft preparation*: Edoardo Bori, Virginia Altamore and Sara Fiore. *Writing—review and editing*: Edoardo Bori, Rachele Saldari, Virginia Altamore, Sara Fiore and Bernardo Innocenti. *Visualisation*: Sara Fiore, Virginia Altamore, and Bernardo Innocenti. *Supervision*: Thomas Luyckx. and Bernardo Innocenti. All authors have read and agreed to the published version of the manuscript.

## CONFLICT OF INTEREST STATEMENT

The authors have no conflicts of interest to declare.

## ETHICS STATEMENT

Our study does not involve human or animal data and requires no ethical board approval.

## Data Availability

The data that support the findings of this study are available from the corresponding author, [BI], upon reasonable request.
